# Addressing Adolescent Substance Abuse and Risky Sexual Health Behaviors via Youth-Led Initiatives: A Review of the Teens Linked to Care Pilot Program

**DOI:** 10.3390/ijerph21030252

**Published:** 2024-02-22

**Authors:** Hailey Bednar, Suzanne McMillan, Turquoise Sidibe, Melissa Bennett

**Affiliations:** CDC Foundation, 600 Peachtree St NE #1000, Atlanta, GA 30308, USA

**Keywords:** Teens Linked to Care, youth-led programming, substance abuse, risky behaviors

## Abstract

The Teens Linked to Care (TLC) pilot program utilized a youth-led integrated strategy to prevent substance use and risky sexual behavior among school-attending youth at disproportionate risk, including sexual and gender minority youth (SGMY). The program developed a framework to address human immunodeficiency virus (HIV), sexually transmitted diseases (STDs), teen pregnancy, and high-risk substance use within schools. Strategies included education, primary prevention, and early detection screening. High schools in two rural counties served as pilot sites and successfully implemented strategies to encourage youth to engage in healthier sexual practices and avoid harmful substance use. An evaluation of TLC demonstrated its effectiveness in developing youth-friendly resources, promoting connectedness, and building resiliency among students and staff. This program used the results of two iterations of the Youth Risk Behavior Survey (YRBS) to understand the situations of youth, including SGMY. YRBS results helped tailor program activities for SGMY populations. By focusing on education, access to care, and supportive environments, schools can utilize the TLC model to combat youth substance abuse and risky sexual practices.

## 1. Introduction

Teens Linked to Care (TLC) was a collaboration between the Centers for Disease Control and Prevention (CDC), the CDC Foundation, and three pilot sites to combat rates of human immunodeficiency virus (HIV), sexually transmitted diseases (STDs), pregnancy, and high-risk substance use among youth in rural schools [1]. Three pilot sites were initially engaged in the TLC program: one per state in Indiana, Kentucky, and Ohio. Throughout the rest of the document, we discuss two of the three pilot sites: Pilot Site A (Indiana) and Pilot Site B (Kentucky). Pilot Site C (Ohio) is not discussed, for in 2019, there was an administration change that resulted in the discontinuation of the TLC program at this site.

### 1.1. Background

Youth engaging in drugs and alcohol have a higher likelihood of engaging in sexual behaviors that put them at risk for other conditions [2,3]. Previous research has identified the link between substance use and sexual activity, but relatively few programs address both behaviors as a connected issue. The TLC program was implemented to understand the occurrence and co-occurrence of such behaviors among youth in high-risk rural communities and to develop programming to address the identified rates among the selected pilot schools [1].

During adolescence, individuals are at particularly high risk for using and abusing substances and subsequently developing substance use disorders, which are associated with higher rates of both physical and mental illnesses and lessened overall health, particularly among teens [2,4]. A number of morbidity rates among youth are attributed to conditions that arise as a result of sexual behaviors, high-risk substance use, and poor mental health [4]. School-based prevention programming targeting risky behaviors offers an opportunity to protect the health of school-attending adolescents.

Many studies have found that sexual and gender minority youths (SGMY), or youth who identify as gay, lesbian, or bisexual or who are attracted to or have sexual contact with people of the same gender [5], are at a higher risk of participating in the use of risky substances and behaviors. A study that evaluated Youth Risk Behavior Survey (YRBS) results from 2015 presented risk estimates for 15 types of substances with risk ratios of LGBQ model-adjusted predicted prevalence [6]. This study found that 51.2% of LGBQ adolescents reported using at least one substance in the past 30 days, and 80.1% reported using at least one substance during their lives. Results presented in this study found that LGBQ adolescents were 1.12 times (95% confidence interval [CI] = 1.06, 1.19) as likely to have used any substance in their lifetime relative to their heterosexual peers and were also more likely to use more than two substances during their lifetime. LGBQ adolescents were more likely to use alcohol, cigarettes, marijuana, methamphetamine, and heroin [6]. It is likely that minority stressors among the LGBTQ population may be at play, leading to such results.

There is a scarcity of programs that address substance abuse and mental health in SGMY populations, and existing programs appear to be insufficient in mitigating inequities faced by SGMY. As such programs are developed and evaluated, best practices should be shared to advance the field and incorporate community perspectives into future intervention development [7].

In 2015, the CDC Foundation and the Centers for Disease Control and Prevention’s (CDC) Division of Adolescent School Health (DASH) collaborated to implement Teens Linked to Care (TLC) as a pilot project.^1^ TLC was an innovative approach designed to address risky behaviors in youth by engaging community-wide support in prevention-related programming. TLC focused primarily on school-attending youth ages 13–19 and encouraged involvement from parents, staff members of community health resources, staff members at the school, and other community members. The population of impact was intended to be youth at disproportionate risk, which included SGMY. The TLC concept focused on the following strategies: health education, access to health services, and safe and supportive environments. These strategies aligned with DASH’s What Works in Schools approach [8]. DASH uses this evidence-based approach to address HIV and STD prevention in schools, but the approach can also be customized to address other health topics, such as adolescent substance use.

Health education provides youth with the information and skills needed to grow into healthy adults; education in schools should cover key health issues encountered by youth and adults alike [9]. There is also a need for personalized resources for SGMY populations in schools. Sexual minority individuals face deficits regarding inclusion in school-based sexual health education, even as SGMY visibility increases [10]. In a study conducted in 2021, adolescent sexual minority males shared that they needed and desired more comprehensive and inclusive sexual health education in schools [11]. As of September 2023, only 10 states require inclusive content in regard to sexual orientation, and 4 states require only negative information regarding homosexuality or positive emphasis on heterosexuality [12].

School-based referral programs can help connect students to youth-friendly healthcare providers and increase adolescent use of health services [13]. Over time, identifying and providing access to youth-friendly health services can lead to a reduction in the burden of morbidities in this age group [13].

Improved education and health outcomes can come as a result of providing safe and supportive school environments, which allow students to better connect with peers and supportive adults in their environment. Providing a safe and supportive environment includes training staff and providing programming that supports youth development, particularly for SGMY populations [3]. Providing youth with a safe environment to learn has direct results in decreasing the likelihood of potential engagement with substances or risky sexual behaviors [3,4]. For SGMY populations, school connectedness has been associated with lower rates of substance use [14,15,16]. Additionally, addressing bullying via in-school policy has been associated with lower levels of substance use, including alcohol [17,18,19].

There is a need for positive social support in schools and communities. Providing a positive social climate for SGMY populations has associated reductions in alcohol and substance use when adjusting for variables such as gender, with lower rates of substance use found among those with access to community resources. This supports the need for joint school–community partnerships in addressing youth substance use [20]. Connectedness between students and teachers leads to positive student behaviors and protection against substance use [21]. Therefore, the goal of TLC was to increase youth knowledge and skills toward prevention of substance abuse and risky sexual behaviors, increase access to youth-friendly healthcare, and provide ways to increase connectedness to communities.

### 1.2. Pilot Site Selection

The first step in TLC setup was determining the location of the pilot programs. The CDC Foundation requested proposals from interested school sites, which were subsequently reviewed by a state site identification committee made up of individuals from state health and education departments. Each pilot site was evaluated against a set of pre-determined criteria: state designation as a rural county, highest substance use and/or HIV/STD rates, prevention capacity, existing programs, and interest in implementing a prevention effort for substance use and sexual health.

Pilot Sites A and B (Indiana and Kentucky, respectively) were both located in designated rural counties and expressed interest in hosting the TLC program. Each site designated concern for youth substance abuse as a 10 on a scale of 1–10 (10 being of highest concern) and expressed concerns related to the misuse of prescription drugs, marijuana, and alcohol. The organizational readiness of each site was also examined for utility in implementing a TLC site. For the purpose of this project, readiness was defined as the capacity of the community to implement programs designed to reduce the likelihood of youth substance use and risky sexual behaviors. Readiness was determined by examining various constructs, including resources, motivation, current prevention collaborations, and other contextual factors. Via the identification of past prevention activities, each site was found to have effective structures in place for implementing an integrated prevention program. The readiness assessment results table utilized to identify pilot sites for TLC can be found in Table A1. Table 1 provides pilot site data that led to their identification.

The chosen pilot sites, despite the identified readiness, had to navigate community stigma surrounding substance abuse and sexual health and had to work to obtain buy-in before the program could be successfully implemented. In the selected rural counties, there was a hesitancy to engage with “packaged programming” as these communities were often targeted for programs implemented without sustainability planning. To address this barrier, the program worked with multiple state-level agencies, such as health and education departments and mental health partners, which allowed for information sharing and promoted community buy-in. The schools and their communities served as the subject matter experts during implementation, as they had the most comprehensive understanding of prioritized needs and available resources. Allowing the school and their community to tailor the program encouraged a strengths-based approach that enabled successful pilot implementation.

State and county requirements for programming around substance abuse and sexual health in schools also posed a barrier for Pilot Site B (Kentucky). For this site, the programming could not be integrated into the school itself; a referral had to be made to a local organization not barred by state requirements to enable programming to occur with a local high school via a planned school–community partnership. This partnership defined the difference between Pilot Site A (Indiana) and Pilot Site B (Kentucky), as Pilot Site A was not barred by state requirements and allowed the program to be implemented directly into the school.

## 2. Methods

### 2.1. Program Strategies

TLC program success relied on the development of Youth Advisory Boards (YABs) and Community Advisory Boards (CABs); community and parent involvement; analysis of program effectiveness; creation of new school curricula; and the implementation and evaluation of the Screening, Brief Intervention, and Referral to Treatment (SBIRT) and Youth Risk Behavior Survey (YRBS). Program activities were centered around improving health education, access to youth-friendly health services, and safe environments regardless of sexual orientation or identity while addressing deficits in student representation and involvement.

A primary aspect of TLC was the youth-focused programming. Schools implemented Youth Advisory Boards (YAB), or groups of school-attending youth, that led the project and made programmatic decisions based on learnings from data and personal experiences. YABs performed a wide array of beneficial activities for the program, including identifying and assessing the local healthcare system for youth-friendly services. They created and disseminated relevant materials related to substance use and risky sexual health and reviewed and modified school anti-bullying policies to be more effective.

As part of the TLC scope of work, each pilot site participated in a youth-led evaluation of their local healthcare providers regarding youth-friendly services. YAB members identified local healthcare providers that addressed sexual health and substance use treatment. Via phone interviews and in-person meetings, YAB members assessed which services were covered and what policies and protocols existed regarding confidentiality. Results were compiled and disseminated to the larger student body at each school to help youth take control of their healthcare needs. These are just a few examples of how the YAB led to successful material creation and dissemination throughout the TLC project.

Programming also involved community members via Community Advisory Boards (CABs) made up of a diverse group of individuals who served as advisors to students from outside the school setting. CABs included representatives from school faculty members, parents or guardians, law enforcement, municipality employees, medical providers, members of local clergy, and former Youth Advisory Board (YAB) members. During the TLC pilot programs, CABs were notably successful in assisting TLC with programmatic planning and implementation.

The TLC core strategies were split into a wide range of activities. The school staff evaluated their health curriculum for substance use and sexual health topics, which resulted in increased training for school staff and the distribution of advocacy-related information to parents and the community. The program assessed in-school referral systems for treatment and addressed barriers, provided training and created resources and plans for movement forward. The program promoted engagement strategies for school connectedness and school health resources and established in-school resource centers.

In response to student-led feedback, addressing bullying became a primary aspect of TLC. The issue of bullying was identified by students as a real threat to their mental health and educational experience. TLC participants worked to establish in-school committees, provide environments for discussion among students, and disseminate materials about the prevention of bullying. To further address bullying, the TLC participants evaluated school safety and identified school affiliates who were, or who could be, trusted adults at each pilot site. Pilot Site A conducted a survey analyzing the current school culture on bullying and how it may be tied to substance use and risky sexual behavior. Results from the survey prompted the creation of an anti-bullying committee in early 2019 consisting of representatives from TLC YAB and school staff. As a result, YAB meeting members voted to begin a campaign that combated bullying by promoting positive self-worth. Another example of YAB activities addressing bullying includes Pilot Site B’s youth-led video campaign, where YAB members developed a script for video production addressing bullying in schools that was aired on the school news station and their community’s local news.

TLC engaged the community at each pilot site and helped foster relationships between students and positive adult figures. The selection of the TLC site coordinators was particularly integral to the program’s success, as this individual became the primary positive adult figure for program participants. Each coordinator exhibited characteristics identified by the students, such as dependability, trustworthiness, being non-judgmental, and serving as a key figure within the community. Some other notable community partners for successful TLC implementation included community organizations, school administrations, local health systems, and local religious organizations.

To increase adolescent knowledge and skills for the prevention of risky health behaviors, TLC supported a new health curriculum at each pilot site’s high school in 2017. TLC staff used a curriculum specialist in CDC’s DASH to evaluate the existing curricula within the pilot site schools. The Health Education Curriculum Analysis Tool (HECAT) was used to compare the existing curricula to National Health Education Standards and CDC’s Characteristics of an Effective Health Education Curriculum. Health educators then participated in a one-day training to review the HECAT results on their existing curriculum and identify a new curriculum that met the above-mentioned standards and characteristics. After assessing gaps, HealthSmart^®^ was selected as the curriculum at both pilot sites because of the emphasis needed on improved sexual health and substance use education [22].

### 2.2. Program Evaluation

Evaluation of the TLC program was performed via monthly reporting and focus groups. Each site utilized monthly reports using the SurveyMonkey platform to keep track of program activities and demographics. During each monthly report, the site leads shared facilitators and barriers to programming that was tracked for progress or resolution. Upon the completion of the program, this feedback was qualitatively analyzed for key facilitators and barriers to program improvement. In the final evaluation, there were 90 final individual monthly report responses from TLC site leads that underwent qualitative analysis. Additionally, focus groups were held after the first year of the program to understand key facilitators and barriers to implementation; key participants included students and school staff who were engaged in the sites’ advisory boards, as these groups engaged in important decision-making for the project. There were 5 total focus groups conducted with Youth and Community Advisory Boards engaged in the TLC program. The focus group guide prompted questions to the advisory boards around a range of topics, including community facilitators, community barriers, program activities, youth engagement, drug use and sexual behavior, and future suggestions.

For both monthly reports and focus groups, the qualitative data collection and analysis process was performed by the TLC evaluation team, which had members from each project partner, including CDC Foundation, CDC, and TLC sites. The steps followed for analysis were similar for each data set: First, sentiment analysis was conducted by conducting a first pass via the data sets, which led to the development of initial codes. Codes and associated definitions were then reviewed by all team members and verified with all project partners. These initial codes were applied to the data sets during in-depth thematic analysis and coding. For both monthly reports and focus groups, qualitative coding was performed by two parties in MAXQDA, each from a different project partner for perspective, and any discrepancies were resolved via collaborative discussions.

One objective of TLC was to increase adolescent access to youth-friendly, key health services by implementing Screening, Brief Intervention, and Referral to Treatment (SBIRT). TLC sites were selected to implement SBIRT, a comprehensive, integrated public health model designed to provide timely referral and treatment for people who have substance use disorders [23,24]. SBIRT implementation provided an opportunity to elevate youth voices in developing an intervention, particularly when it comes to the lived experience of youth engaging with substances. Providing culturally appropriate care can improve outcomes for populations disproportionately impacted, including SGMY groups [25]. Implementing SBIRT among school-attending youth allowed for a better understanding of the use of SBIRT with youth, as SBIRT is primarily implemented among adult populations. SBIRT was evaluated via the evidence-based method [23,24].

The Youth Risk Behavior Survey (YRBS) assesses health-related behaviors among youth, including those that the TLC program was working to address [4]. YRBS was utilized to evaluate TLC implementation. TLC selected site-specific YRBS questions about substance use and risky sexual behaviors. All students who were present at the schools during YRBS dissemination days were given the opportunity to participate in the survey if granted passive parental permission. Similar schools in each pilot site’s respective areas were chosen to also implement the YRBS survey as comparative controls. Control sites for YRBS survey implementation were chosen based on proximity to their comparison pilot site; each control site was within 5 miles of their comparison pilot site. This allowed for the closest similarities for state and county context. The survey was conducted twice with TLC sites and control schools, first in 2019 and again in 2021 [26,27]. During analysis, a weight was added to each questionnaire to reflect the likelihood of sampling each student and to reduce bias by compensating for different patterns of nonresponse. The weight used for estimation was W = f1 × f2, where f1 = a student-level nonresponse adjustment factor calculated by class, and f2 = a post-stratification adjustment factor calculated by gender within grade.

It is important to note that while SGMYs were a focus of the research, these youth were not a focus of the intervention itself; the intervention focused on safe and supportive environments for all youth regardless of gender, race, ethnicity, or sexual identity. The SGMY population-related results are reflected in the outcomes of interest for YRBS data that were collected.

## 3. Results

While strategies used by the TLC program were not specific to SGMY populations, it was important to consider related outcomes if the data were available, particularly when it is known that disparities in sexual health and substance use are higher in SGMY populations. Multiple evaluation approaches were identified to help better understand the needs of program recipients and to yield more accurate recommendations to enhance program development and change.

### 3.1. Programmatic Reporting and Focus Groups

Programmatic reporting was collected between April 2019 and March 2022; each site provided input on each monthly report regarding catalysts and roadblocks to programmatic success. These reports were provided on a monthly basis to ensure a continuous feedback loop. Upon collection of each monthly report, themes were input into an internal programmatic tracker and monitored throughout the program.

The following barriers were identified as priority items that may limit the overall implementation of the program: time available, location and transportation, school administration, competing school activities, student representation, and support from adult community members. Themes were also identified to further understand student engagement and representation in the program. Barriers to student engagement identified by sites included meeting logistics and operations (time and location), other priorities (school or external), transition to virtual education during COVID-19, and lack of engagement from parents. Activities identified as cultivating student engagement included providing increased opportunities for engagement with the community, promoting activities of interest to youth, providing flexibility in meeting times and spaces, hosting events outdoors and increasing promotion of the program at the school.

TLC program participants within the YAB and CAB also engaged in a total of five focus groups to discuss major facilitators and barriers to implementation. The biggest barrier that was identified across both pilot sites was community stigma. TLC CAB members shared that students do not often share their honest experiences with substances, as they fear the repercussions if they are caught. In the identified communities, CAB members reported that there is a community standard of not seeking help for substance abuse disorders, which affects the perceptions of the younger generations. Additionally, members of both the YAB and the CAB shared that there were limited youth-friendly resources for advice and education about substance abuse and sexual health, which prevented health-seeking behaviors. It was also noted by a TLC-involved CAB member that since the focus of many resources is preventing substance abuse in adults, it is much more difficult for youth to find similar resources related to them. TLC program leads shared that it is taboo to discuss sexual health in the school setting, which limits feedback that students share regarding the behaviors that they engage in. These barriers were overcome by emphasizing the youth-led approach that allowed them to understand the data and make decisions on what programming can improve the health of their peers.

The TLC program coordinators at each site were identified as the primary facilitators for the success of each program; internal support between students and supportive adults was necessary for program success and helped to catalyze discussions that dismantled stigma within the schools. As the pilot established the necessary positions to implement the program, it was determined that the biggest need was for a program site coordinator who would serve as a supportive adult. This individual would need to maintain the youth-led approach of the program and guide the students in decision making. This adult needed to be integrated into both the school and community, be trusted by students and their parents, and have an understanding of substance abuse and sexual health. Student participants in TLC shared in focus groups that the program coordinator should be dependable, nonjudgemental, and respected among their peers.

### 3.2. Screening, Brief Intervention, and Referral to Treatment (SBIRT)

For the TLC program, SBIRT data were not stratified by sexual orientation or gender. SBIRT during the TLC program followed the following process: screening via the SBIRT Check Yourself web-based questionnaire, a confidential survey filled out by community members and the school facility; brief intervention immediately following the screening, typically 10–15 min; and referral to treatment. SBIRT was conducted using the youth-friendly service provider resource during the “referral to treatment” segment. During SBIRT implementation at the pilot sites, 61 students voluntarily self-screened (35% of the student population) via the Check Yourself web survey. Based on self-screening results, students received brief interventions if it was deemed necessary; 17 students received brief interventions (25% of screened students), and 15 students were referred to treatment (25% of screened students). Nationally, the average number of students screened who are then referred to treatment using SBIRT is 5%. Due to COVID-19, the SBIRT administration did not continue during the project period as the healthcare providers were no longer providing services in person, and it became challenging for students to seek help safely and confidentially in their community.

### 3.3. Youth Risk Behavior Survey (YRBS)

YRBS data supported the implementation of improved health education for encountering substances or risky sexual behaviors, particularly among SGMY populations. For pilot site YRBS implementation, measures were adjusted to reflect relative risk in relation to a percentage of students among a class of 30 in order to make results relatable to the average classroom size. YRBS data were used by YABs and CABs to address areas of concern for programming for the coming year. Both administrations of the YRBS survey in 2019 and 2021 included comparison results to nearby control schools [25,26]. Response rates for each site are found in Table 2. Survey respondents provided demographics, including sex, race/ethnicity, sexual identity, and sexual contacts (Table 3).

Demographic differences between pilot sites and control sites can be attributed to differences in the relative populations of each county where the schools sit. The relative distribution of student demographics, when compared to the demographics of the community in which they reside, maintains similar trends. Notable differences in demographics for YRBS implementation included the racial distribution of the student body attending Pilot Site B; while Pilot Site B had a minority rate that was much higher than those in the other rural areas chosen for the TLC program, it should be noted that this had no bearing on why they were chosen as a site. The program was not intended to be implemented in schools with specific demographics; rather, the program was intended for rural schools that sit within a county that was encountering high rates of substance abuse and HIV/STD during pilot site selection.

While limitations exist in comparing data between two YRBS years, such as changes in school staffing, student samples, and the COVID-19 pandemic, Pilot Sites A and B had improved relative measures for the rates of students who engaged with risky substances and behaviors. In the analysis process, each risky behavior identified on the YRBS survey was stratified by the demographics shared in Table 1, including race/ethnicity, sexual identity, and sexual contacts. Engaging in this process allowed for a further understanding of behaviors that specific subpopulations at each school engaged with to determine whether a more targeted approach was needed. Data analysis identified significant differences in risky behaviors faced by those identifying as SGMY. Comparing participation rates in risky behaviors between heterosexual and gay, lesbian, or bisexual students that attended each pilot site helped to initiate programming at the school that targeted specific concerns and to provide a more well-rounded program to all students that participated in the program.

The TLC YRBS study found similar results to the Caputi 2018 study [6]. Each of the pilot sites was able to compare data from the heterosexual student population with the SGMY (gay, lesbian, or bisexual) student population to understand differences. Students who identified as gay, lesbian, or bisexual had a higher likelihood than their heterosexual counterparts to engage in cigarettes, alcohol, marijuana, cocaine, inhalants, heroin, methamphetamines, and ecstasy. The only case where heterosexual counterparts were more likely was when cigarettes were smoked frequently or on 20 or more of the 30 days that occurred before answering the survey (Table 4). For both pilot sites, between 2019 and 2021, the percentage of respondents who identified as gay, lesbian, or bisexual that used cigarettes, alcohol, marijuana, cocaine, inhalants, and methamphetamines decreased. A decrease in the use of heroin and ecstasy by respondents who identified as gay, lesbian, or bisexual occurred at Pilot Site B. Those who identified as heterosexual also had relative improvements between 2019 and 2021 in cigarette smoking and the use of marijuana, cocaine, heroin, methamphetamines (one pilot site), smoking cigarettes frequently, inhalants and ecstasy (both pilot sites) (Table 4). While these data are not a direct reflection of the program’s success, they display how some prioritized risky behaviors decreased during the program, potentially as an indirect result of activities that were implemented.

Anti-bullying policies and programs are commonly associated with lower substance use in SGMY populations [12]; between 2019 and 2021 YRBS data, bullying decreased across all students, heterosexual or gay, lesbian, or bisexual (Table 4). The anti-bullying campaign was a potential contributor to this, as was the project that YABs engaged with to understand their peers’ experiences with bullying within the school setting. Further discussion regarding the comparison between pilot and control site SGMY data can be found in the limitations.

## 4. Discussion

TLC was an innovative project that integrated reducing substance use and risky sexual behavior into one project. It included various strategies and activities based on best practices and guiding principles in substance abuse prevention and sexual risk reduction. The TLC youth-led initiative was successful in engaging the community in reducing risky behaviors and substance abuse, as evidenced by youth engagement in SBIRT, quantitative data from YRBS, and qualitative statements collected throughout the duration of the program. Representatives from the YAB shared in programmatic reporting that there was a desire for increased resources related to the prevention of substance use, as they had learned that it was easier to prevent than to treat.

YRBS showed that risky behaviors were occurring among students at each site, including the use and consumption of dangerous substances. Many students from each of the sites were sexually active. The co-occurrence of these activities provided urgency in the schools to implement the TLC programmatic activities to address these public health challenges. The comparison of the pilot sites to control sites showed that similar schools in the same areas of TLC pilot sites had similar risky behaviors. The pilot sites for TLC demonstrated a decrease in some identified risky behaviors, such as the use of cigarettes, alcohol, marijuana, cocaine, inhalants, and methamphetamines (Table 4). It is important to note for these data that different YRBS years are made up of different samples as student composition at each school changes from year to year, but the relative improvements between the 2019 and the 2021 data sets provide a starting point for future TLC implementations seeking to address similar risky behaviors.

Acting on a foundation of sensitivity was at the forefront of programmatic implementation, as there are particular environments that put up walls for implementing programs related to sexual health. Because a requirement for site selection included increased HIV rates, it was vital to understand the community norms and attitudes surrounding the risky behaviors. Familial and community norms led to pervasive stigmas, often leading to limited integration of topics into health education. TLC program staff identified in reporting that youth are afraid to open up about these topics because they want to avoid getting in trouble. TLC staff also reported that community stigma was a huge barrier to implementation, as many within the community did not seek help. To successfully implement a sexual health program, buy-in must be obtained by leaders of the community and organizations that may need to be involved.

Programmatic success and sustainability additionally relied on promoting community buy-in and engagement. Involvement from individuals in TLC CABs across multiple sectors, including medical services, social services, and public safety, helped to garner the necessary resources at pilot sites to have the ability to continue the program beyond allocated funding. Such relationships allowed for necessary discussions to occur across the community to ensure proper buy-in and support, helping to reduce the stigma surrounding a school-based sexual health and substance misuse education program. At both pilot sites, CAB members were willing to donate time, services, space, food, and other program needs to TLC to maintain it beyond grant periods. Support and engagement from community leaders are necessary for the long-term success and sustainability of the TLC program.

Overall, it was found that one of the primary reasons for programmatic success was the building of trust between the students and the adults involved in the program. Providing youth with leadership roles and decision-making opportunities was necessary for ensuring positive engagement with programmatic implementation, which was primarily carried out via the YAB and CAB relationship. The foundation of TLC’s success can be attributed to the collaboration between students and supportive adults and the opportunity for youth voices to be heard in implementing programming within their environment.

## 5. Conclusions

TLC activities provided health education via initiatives and youth-led activities, access to essential health services via the referral program, and reinforced supportive environments. This pilot youth-led program showed success in increasing youth referred-to care, education for youth and parents, and community support for reducing risky behaviors, demonstrating that this youth-led model warrants further implementation and examination in rural school settings. While the program itself was not tailored to SGMY populations, it is important to understand how this program affected their school experience, and the YRBS data helped provide a platform for advocacy and action. Continued research is needed to understand the effects that similar programs have on youth in schools and specifically how these programs affect SGMY that are engaged.

## 6. Limitations and Next Steps

This research reflects two successful implementations of a youth-led program addressing substance misuse and sexual health. Given the uniqueness of these projects and communities, it is uncertain whether similar types of success can be anticipated at other locations. There is a need for continued research into youth-led programs in schools to address substance misuse, sexual health, and mental health. Additionally, the next steps can include further research into the sustainability of youth-led programs addressing highly stigmatizing topics, including how to address such stigma in the community and how youth-led approaches can help to address related barriers to implementation.

Comparing YRBS data has limitations as there are different populations of students responding. Additionally, both administrations of the survey were taken in different contextual situations, as the 2021 survey was administered after the COVID-19 pandemic. While this led to decreased student engagement in school activities due to a transition to virtual learning, it could have also had an effect on the outcomes of YRBS, as it is possible that during the COVID-19 pandemic, there was less opportunity for the study population to engage in sexual and substance use behaviors. While comparing the data does not prove the success of TLC in addressing such issues for SGMY, it does prove the high importance of such programs to improve health education and substance abuse programs specifically for students who identify as SGMY.

Additionally, YRBS data analysis posed limitations due to the limited SGMY sample size. In this case, data stratified by sexual identity could not be compared between pilot sites and control sites. YRBS data analysis, upon being weighted by the identified estimation calculation, led to the inability to provide percentages to groups with suppressed response rates; therefore, for data listed for Control Site A and Control Site B, a “-“ indicates that there were fewer than 30 students in the denominator (subgroup) and/or fewer than 10 students in the numerator. This also limits the ability to compare Control Site data between 2019 and 2021. Therefore, more surveillance studies should be conducted with the identified populations across multiple sites to further understand the effects of school-based differences across SGMY groups.

The TLC program did not directly address and was not tailored to SGMY populations for reasons explained. Despite this, data collected and feedback shared helped participating students understand the conditions of their peers and provided them the motivation needed to help create a more inclusive and accepting youth-friendly health program. There should be further actions to tailor similar programs in the future to help subpopulations of students who experience heightened disparities in health access and education, and there should be further research on how such programs affect SGMY populations. A toolkit for implementing the TLC program, created in partnership between the CDC, CDC Foundation, and TLC pilot sites, can now be referenced on the CDC’s Teens Linked to Care webpage (https://www.cdc.gov/healthyyouth/substance-use/teens_linked_to_care.htm (accessed on 1 July 2023) [1].

## Figures and Tables

**Table 1:** Pilot site descriptions and demographics at time of selection (2015).

	Pilot Site A (Indiana)	Pilot Site B (Kentucky)
Organization	High School Program	High School Program Managed by Community Organization
Rural County	Yes	Yes
2015 Population	23,744	90,366
Unemployment ^a^	6.7%	5.2%
Children in Poverty ^b^	27%	19%
Rate Uninsured ^c^	16%	14%
High School Graduation Rate ^d^	76%	92%

^a^ Unemployment is the percentage of the population ages 16 and older unemployed but seeking work [8]. ^b^ Children in Poverty is the rate of people in the population under the age of 18 living in poverty [8]. ^c^ Rate Uninsured is the percentage of the population under age 65 without health insurance [8]. ^d^ High School Graduation Rate is the percentage of adults ages 25+ with a high school diploma or equivalent.

**Table 2:** YRBS response rates for TLC pilot sites and control sites in 2019 and 2021.

Year	Pilot Site A	Control Site A	Pilot Site B	Control Site B
2019	90%	78%	78%	80%
2021	82%	94%	75%	71%

**Table 3:** YRBS-reported demographic information for TLC pilot sites and comparison sites for 2019 and 2021 [25,26].

YRBS Demographic	Year	Pilot Site A (Intervention)		Control Site A (Control)		Pilot Site B (Intervention)		Control Site B (Control)	
		%	N	%	N	%	N	%	N
Sex: Female	2019	*52.7*	*145*	46.3	42	45.9	127	48.7	77
	2021	*57.5*	*143*	42.4	48	*47.4*	*109*	39.5	84
Sex: Male	2019	47.3	139	*53.7*	*49*	54.1	132	51.3	83
	2021	42.5	102	*57.6*	*61*	52.6	111	*60.5*	*88*
Age: 15 or younger	2019	*41.2*	*130*	30.8	28	49.7	128	46.9	86
	2021	42.1	105	39.1	46	49.4	106	48.3	96
Age: 16 or 17	2019	48.9	133	52.4	48	43.5	119	*48.4*	*68*
	2021	52	129	53.2	55	45.8	109	46	74
Age: 18 or older	2019	9.9	22	*16.8*	*15*	6.8	15	4.8	7
	2021	5.8	15	*7.7*	*9*	4.8	10	5.7	5
Race/Ethnicity: Black	2019	0.6	2	*2.2*	*2*	*19.3*	*48*	2.6	5
	2021	*0.9*	*3*	0	0	*20.3*	*38*	3.8	7
Race/Ethnicity: Hispanic/Latino	2019	4.8	14	4.3	4	*18.1*	*43*	13.9	23
	2021	*7.6*	*18*	5.2	6	*18.8*	*48*	11.5	19
Race/Ethnicity: White	2019	*91.7*	*255*	81.9	73	49.5	129	*76.9*	*118*
	2021	88.7	214	86.7	91	45.1	95	*77.3*	*128*
Race/Ethnicity: All Other Races	2019	1.4	4	*6.7*	*6*	*2.4*	*6*	1.2	2
	2021	0.8	2	*1.7*	*2*	1.9	3	2.1	4
Race/Ethnicity: Multiple Races	2019	1.4	4	*4.8*	*4*	*10.8*	*26*	5.5	10
	2021	2.1	4	*6.4*	*7*	*13.9*	*29*	5.2	10
Sexual Identity: Heterosexual	2019	84.1	235	*87.7*	*80*	77.0	189	76.8	121
	2021	70.2	166	*81.1*	*86*	63.6	131	67.2	114
Sexual Identity: Gay, Lesbian, or Bisexual	2019	*11.8*	*33*	9.2	8	18.9	46	16.3	26
	2021	*23.1*	*55*	9.9	11	17.2	35	19.9	34
Sexual Identity: Not Sure, Other/Questioning	2019	4.1	11	3.1	3	4.2	10	6.9	11
	2021	6.8	16	8.9	9	19.3	40	12.9	22
Sexual Contacts: Opposite Sex Only	2019	42.4	113	46.4	39	41.7	91	*46.7*	*70*
	2021	40.8	90	41.1	42	35.7	65	36.3	55
Sexual Contacts: Same or Both Sexes	2019	10.0	27	8.0	7	12.9	28	10.7	16
	2021	13.6	30	12.8	13	9.1	17	*14.2*	*22*
Sexual Contacts: No Sexual Contact	2019	47.6	127	45.7	38	45.4	99	42.6	63
	2021	45.6	101	46.1	46	55.2	101	49.6	75

Italicized cells represent significant differences between pilot and control site demographics determined via *p* < 0.05 comparison performed via *t*-test. N = The number of students in this subgroup = fewer than 30 students in the denominator (subgroup) or less than 10 in the numerator.

**Table 4:** YRBS data from 2019 to 2021 regarding chosen risky behaviors between Pilot Site A, Control A, Pilot Site B, and Control B for chosen sexual identities (heterosexual and gay, lesbian, or bisexual).

Substance Use Risk Behavior (YRBS Question)	Year	Pilot Site A (Intervention)		Control Site A (Control)		Pilot Site B (Intervention)		Control Site B (Control)	
		Heterosexual (%, N)	Gay, Lesbian, or Bisexual (%, N)	Heterosexual (%, N)	Gay, Lesbian, or Bisexual (%, N)	Heterosexual (%, N)	Gay, Lesbian, or Bisexual (%, N)	Heterosexual (%, N)	Gay, Lesbian, or Bisexual (%, N)
QN32: Percentage of students who currently smoke cigarettes (on at least 1 day during the 30 days before the survey)	2019	7.7, 231	*23.8, 31*	9.1, 76	-, 6	2.6, 179	*18.9, 40*	10.1, 120	-, 25
	2021	3.1, 166	*13.2, 49*	9.0, 86	-, 11	4.4, 131	*10.9, 35*	2.7, 107	*16.9, 35*
QNFRCIG: Percentage of students who currently smoke cigarettes frequently (on 20 or more days during the 30 days before the survey)	2019	2.4, 231	*8.1, 31*	1.2, 76	-, 6	0.0, 179	*1.9, 40*	1.9, 120	-, 25
	2021	0.7, 166	*8.2, 49*	1.3, 86	-, 11	0.0, 131	*4.7, 35*	0.0, 107	3.5, 35
QN41: Percentage of students who currently drink alcohol (at least one drink of alcohol on at least 1 day during the 30 days before the survey)	2019	15.3, 226	*37.7, 30*	28.4, 71	-, 9	12.1, 164	*30.2, 41*	21.9, 116	-, 25
	2021	17.7, 158	*25.6, 50*	23.0, 82	-, 9	13.7, 125	*25.9, 32*	27.6, 100	*38.0, 36*
QN47: Percentage of students who currently use marijuana (one or more times during the 30 days before the survey)	2019	10.3, 234	*33.5, 32*	16.5, 78	-, 7	14.7, 184	*41.8, 41*	16.9, 123	-, 25
	2021	9.6, 168	11.3, 47	28.3, 82	-, 11	16.7, 130	*32.2, 34*	16.5, 106	*32.1, 35*
QN50: Percentage of students who ever used cocaine (any form of cocaine, including powder, crack, or freebase, one or more times during their life)	2019	1.2, 233	*9.3, 34*	2.4, 78	-, 9	3.1, 181	*16.1, 48*	2.2, 122	-, 24
	2021	2.9, 164	*7.4, 49*	1.1, 83	-, 11	2.5, 128	5.1, 36	7.5, 107	13.3, 37
QN51: Percentage of students who ever used inhalants (sniffed glue, breathed the contents of aerosol spray cans, or inhaled any paints or sprays to get high one or more times during their life)	2019	4.7, 234	*19.4, 34*	5.9, 78	-, 9	4.4, 184	*21.6, 49*	5.3, 121	-, 25
	2021	4.0, 165	*21.3, 49*	4.7, 83	-,11	3.4, 129	*10.3, 37*	6.7, 108	*25.7, 37*
QN52: Percentage of students who ever used heroin (also called “smack”, “junk”, or “China White”, one or more times during their life)	2019	*0.4, 234*	0, 34	1.2, 77	-, 9	3.0, 184	*14.0, 49*	0.0, 122	-, 25
	2021	0.6, 165	*7.4, 49*	0.0, 83	-, 11	0.5, 129	*7.5, 37*	2.6, 108	*13.3, 37*
QN53: Percentage of students who ever used methamphetamines (also called “speed”, “crystal meth”, “crank”, “ice”, or “meth”, one or more times during their life)	2019	0.8, 226	2.5, 34	0.0, 77	-, 9	3.6, 179	*19.0, 48*	0.0, 120	-, 25
	2021	1.3, 164	1.5, 49	0.0, 81	-, 11	0.5, 126	*10.2, 36*	2.4, 207	*18.7, 37*
QN54: Percentage of students who ever used ecstasy (also called “MDMA” or “Molly” one or more times during their life)	2019	*1.3, 234*	0, 34	2.4, 78	-, 9	2.6, 183	*20.5, 49*	2.1, 122	-, 25
	2021	2.4, 165	*12.7, 50*	3.5, 83	-, 11	1.9, 129	*7.1, 36*	7.7, 108	12.4, 37
QN23: Percentage of students who were bullied on school property (ever during the 12 months before the survey)	2019	24.0, 235	46.7, 33	25.4, 79	-, 9	15.2, 184	51.3, 47	24.2, 123	-, 25
	2021	16.7, 168	31.5, 48	11.9, 86	-, 11	8.5, 131	27.5, 37	22.7, 109	27.8, 38

Cells identified with italicized text are risky behaviors for which the sexual identity (heterosexual or gay, lesbian or homosexual) has a statistically significant difference in identifying with the risky behavior based on a *t*-test analysis, *p* > 0.05. Statistical significance is not comparative between sites. N = The number of students in this subgroup. N = number of students in the subgroup that answered = fewer than 30 students in the denominator (subgroup) or less than 10 in the numerator.

## Data Availability

Data can be shared upon request to the corresponding author.

## Appendix A

**Table A1:** Pilot readiness assessment results table.

	Location A	Location B	Location C
How much of a concern is youth substance use on a scale of 1–10?			
What is the most pressing substance abuse issue in the community?			
Community efforts in place to address substance use?			
How much of a concern is youth risky sexual behaviors on a scale of 1–10?			
Key Community Organizations/Agencies			
